# Sentience and transcendence: personal reflections on physical and mental adaptation from a near-death event and life-limiting surgery

**DOI:** 10.1192/bjb.2020.137

**Published:** 2021-06

**Authors:** James Ramsay

**Affiliations:** Diocese of Norwich, Church of England, UK

**Keywords:** Sentience, transcendence, wholeness, person-centred, near-death

## Abstract

In this article I reflect on my experience of adapting physically, mentally and spiritually to a medical trauma that had life-changing consequences. I consider how, over 7 years to the time of writing, mental difficulties were inseparable from the physical; and how, for me, both are aspects of a form of understanding knowable only as mystery. Writing from a position of religious faith, I try to convey my experiences in a way that will be of interest to others regardless of their views. At the end, I reflect on aspects of my care that might be particularly relevant for a holistic, person-centred therapeutic approach.

In 2012, while working as a vicar in Newham, East London, I suffered an ‘abdominal catastrophe’ necessitating emergency resection of most of my small bowel and a portion of colon. I now live on intravenous nutrition (TPN, total parenteral nutrition, fluids and chemicals pumped direct into the bloodstream through a tube ‒ in my case a Hickman line ‒ permanently inserted into a central vein, thus bypassing the digestive system). The crisis occurred out of the blue. I was 60 years old and in good health.

On the way from Accident and Emergency to be prepared for theatre I had a cardiac arrest, triggered, it was subsequently concluded, by disruptive electrical impulses owing to bowel ischaemia. I was revived after 6 minutes of cardiopulmonary resuscitation (for days after the operation I was puzzled why, in addition to the pain in my abdomen, my chest hurt so much). From a side room, as she was being warned of a not very hopeful outcome, my wife Celia saw me being wheeled in an oxygen tent for a second computed tomography scan.

The operation was completely successful, and physical existence felt near-miraculous as I came out of the crisis. Over the following months and years, however, as TPN became normality, my memory of the trauma lost much of its emotional resonance and life began to feel as mundane as if nothing had happened. Faith sustained me through physical challenges and depressive periods, return to work and early retirement, but I found myself asking age-old questions: Is there meaning to all this? What is it to ‘live’ rather than merely ‘exist’? How can life be at once so cruel, so tedious, so beautiful? Far from abstract, this questioning was visceral: gut experience (recognising the irony of the image) striving for conceptualisation. Pre-catastrophe faith had to engage with both a new wilderness of meaninglessness within myself and a baffling sense of transcendent blessedness ‒ experiential encounter with the teaching (e.g. *Mark 8.35*) that we must lose our life to find it, a maxim I return to later in this article.

## The anaesthetist and the chaplain

As I was being prepared for theatre, consciousness was a floating state anchored to a locus of morphine-dulled pain I knew to be my body. ‘I’ was an internal voice observing, joking about signing (scrawling, on my back) the consent form in such circumstances, and instructing Celia where the church wardens would find the service sheets I had prepared for Christmas in 4 days’ time.

The anaesthetist chatted to put me at my ease.

‘What do you do?’ she asked. ‘Your job?’

‘I'm a vicar.’

‘That's nice.’

‘Not everyone thinks so.’

‘Well, I do! I'm Orthodox.’

I tried to sit up, but movement was no longer possible. It was the beauty of the Eastern Orthodox Liturgy that, years before, had attracted me back to Christian faith.

My next recollection is of the anaesthetist saying, close beside me,

‘I'm very worried.’

‘I know.’ From the outset she had voiced anxiety about the situation.

‘They've told you this is a major operation. But I don't think they have really … You do realise, don't you? It is … very serious.’

The rhetorical question and understated comment were exactly what I needed: they enabled my instinctive knowledge that I might die to surface fully into consciousness. Our earlier exchange had (I think) permitted her to follow her hunch that, although ‘the patient’ must not be distressed, ‘the priest’ would wish to be alerted to the prospect of dying.

‘Can I see a chaplain?’ I asked.

The on-call chaplain was Brother Julian, a Franciscan who ran a local hostel for homeless people, and whom I knew well. His usual beaming smile brought into the room the whole universe of faith ‒ in which pain, fear and death are embraced within an infinitely vaster continuum of grace, compassion and joy. There was neither time nor need for words beyond thanks, prayer and a blessing. Having come straight to the preparation room, Brother Julian offered to fetch the oil of anointing from the multifaith room the other end of the hospital, but I heard Celia say, ‘Can we get on?’ and I was happy with this. Were we not ‘one flesh’? Everyone then withdrew for Celia and me to say our maybe/maybe-not goodbyes. I entered sedation in a state of great peace and awareness of love, confident that whichever way the operation went, it was toward life.

## Religious experience, faith and spiritual context

A conventional English private education had ensured I knew my Bible and Book of Common Prayer. At home, however, although there was no active hostility to faith, religion had not been part of life. At university I became gripped by theology, but almost as if ‘I’ were another person: real spiritual life was art, music and pleasure, with a lazy inclination toward Taoism. Then, at 27 years of age, I had an overwhelming inner experience that rid me of suicidal fantasies I had entertained since adolescence. Although disliking the phrase ‘born again’, which I associated with people who seemed stuck in their conversion experience, this event felt indeed like re-birth, in line with Jesus's dictum: ‘I tell you, unless someone is born from above, he cannot see the Kingdom of God.’ (*John 3.3, The New Testament, A Translation* [David Bentley Hart]).

Spiritually I felt most at home in the mystical tradition of patristic and Orthodox writers, The Cloud of Unknowing and St John of the Cross. The theology of the Vedas and traditions of contemplative prayer in all faiths brought spiritual enrichment. Denominational Christianity seemed cramping. But hearing my default Anglicanism described somewhere as ‘the most spacious mansion in Christendom’, that is where I committed myself.

Ordained at 34 years of age, I had served most of my ministry in institutionally marginal parishes with wider community commitments: in Newham, these included work with refugees; and when I was in Accident and Emergency, stories of people elsewhere in the world suffering atrociously without medical support played in my mind like a kind of mantra, sustaining me in my own lesser experience of pain and powerlessness.

## Short bowel syndrome, TPN and therapeutic context

It transpired my abdominal catastrophe had been caused by a section of small bowel becoming trapped in a caecal hernia, of which I had been unaware, starving my whole gut of oxygen. Yet this functionally useful explanation (no further investigations needed) did not satisfy my hunger for an explanation at a holistic rather than instrumentalist level.

Through parish work and ministerial training, I was acquainted with the National Health Service at every grade and had visited nearly every kind of hospital ward. A patient's view is radically different. Nevertheless the Gospel imperative to visit the sick had prepared me in the sense that, through empathy and compassion, however partial, I brought better informed awareness to the experience.

It took time to get the hang of TPN. I now infuse 6 days a week, for 14 hours each infusion. The aseptic procedure for connecting and disconnecting is fiddly and time-consuming. Of several side-effects, the most immediately disagreeable and socially awkward is constant diarrhoea ‒ despite having virtually no appetite, I have to eat to maintain other organ functions (my surgeon had expressed pleasure that, with still just enough jejunum to connect to my colon, I did not need a colostomy).

Thankfully I can walk about with pump and fluids in a backpack during infusions. However, with the functions of physical existence taking up so much time, every day I face the question of what makes existence *meaningful*. In that confrontation faith is a compass and map, as well as emotional sustenance, toward an answer that (as, since my conversion, I no longer give credence to the existentialist Absurd) must necessarily be greater than existence: God, obviously. Yet the word ‘God’ has become so commodified it is ever more incapable of bearing the weight of its own meaning.

From theological college I had gained outline familiarity with psychotherapeutic theories and praxis, and over the years had myself undergone two periods of counselling in relation to stresses present and past. I also regularly talked with a spiritual director. In the parish I offered a listening ear to people going through difficult times, some of whom found the feelings stirred up in clinical therapy sessions hard to handle (my role being to support a parishioner, not intrude upon therapeutic ground). However, in the aftermath of abdominal catastrophe I needed first and foremost to recover a sense of myself simply as a human being, rather than an *object* of surgical, therapeutic, caring or technological intervention.

Only gradually did I come to recognise this feeling of being less than fully human. Poor communications across the National Health Service, management muddles and homecare company inefficiency compounded mental stress. Lacking strength to ‘think positive’, however, I rediscovered a sense of agency through silent, often wordless prayer. Acknowledging Christ in all I met, from consultants to cleaners and names at the bottom of emails, I regained a sense of humanity and joy. Holding prayerfully in mind the millions around the world enduring infinitely worse, I outfaced the petty humiliations of dependency.

Curiosity about my condition helped combat spiritual stasis and temptation to self-pity. Although not disabled, I have lost an essential organ. A plastic tube sprouts from my chest. I receive artificial nutrition. Certain activities are now problematic or impossible: risk management is self-conscious, psychological nervousness or cavalier overcompensation hard to avoid. After two bouts of septicaemia (the second time, going into septic shock within hours), I agonise about carelessness. Having also suffered nerve damage in my right hand and developed atrial fibrillation and low thyroid on top of normal ageing problems like arthritis, my body clamours for attention. Yet all remind me … I am ALIVE!

## The body as a spiritual organism

Bodily demands are depressing; at the same time, off the flint of faith, so to speak, they spark amazement (as in ‘amazing!’, ‘wow!’ or religious ‘Amazing Grace’; but also an inner maze of numinous darkness, struggle and trust, wilderness and promise). Christianity proclaims ‘the Word’ ‒ the cohering principle of the cosmos ‒ revealed in the uniqueness of an individual historically existent human being. Incarnation, sometimes termed ‘the scandal of particularity’, defining Jesus as both human and divine, makes for a faith in which at one level the spiritual is set aside. In the mystical tradition particularly, the body can become the entire focus of the transcendent.

Vital to my progress was and is contemplative prayer: a spirituality, sharing similarities of practice with other faith traditions, in which the body is not merely a transient vehicle for spirit but, in its very mortality, a ‘temple’ (*I Corinthians 3.16*) of glory, of the eternal nature and identity of the divine indwelling time. Jesus tells his disciples that to find life they must lose their lives for his sake and for the sake of the ‘good tidings’. The Greek word translated as ‘life’ here is *psyche*: meaning not only body (*soma*), but also intelligence, imagination, feeling, intuition, consciousness, all that constitutes personal identity ‒ soul (somewhat like Hebrew *nephesh*, not disembodied Neo-Platonic soul).

My catastrophe made me experientially aware of my disposability. However, survival brought an intimation, equally intense, of the transcendent *soma pneumatikon*, the resurrection body of divine ‘breath’ or spirit, *pneuma* (*I Corinthians 15*). Insofar as faith is an owned creaturely experience, diverting one, at the level of will, from unconditional openness to the love of God, I realised that losing one's *psyche* entails losing faith itself.

## Near-death experience and sentience

My cardiac arrest was accompanied by no near-death experience in the normal sense of extraordinary feeling or vision. Such experiences inspire considerable popular and research interest. However, any near-death event (most people who recover from a near death event do not report a near-death experience) raises important questions about human *being* at a liminal juncture.

At a brain-conscious level there may be nothing; yet our being in its wholeness is more than consciousness: the fact of existence is inseparable from individual and communal *identity*. As a priest, I find it significant that a body before burial or cremation is (to relatives and friends) still ‘her’ or ‘him’ rather than ‘it’. This reflects the subjective emotions of the living; yet at the same time, disposal of human remains has archetypal cultural significance, suggesting that a human corpse cannot be totally reified without violation of some quality essential to human being. Can that quality be pinned down?

My 6-minute outage from normal existence is a blank to me. However, my sense of who I am also has an unconscious relational, narrative dimension. My consultant tells me that the physiological effects of cardiac arrest are not easily identifiable, and personal realisation of what I had been through had a psychological effect on me. The event of which arrest was part had, in its wholeness, a drama incommunicable through empirical analysis. Comprehension (‘grasping together’) requires comprehensive assimilation: epistemic integration of experience at every level, including that of the basic organic matrix of existence, the fundamental conditionality of experience.

I had not been dead for 6 minutes, but what had I been? ‘Near death’ sounded banally quantitative, avoiding qualitative definition. ‘Clinical death’ was portentous, but did not help me understand. ‘Understanding’ would require, I felt, not merely an empirical cognitive account, but some sapiential *event* at the level of my whole being, resonant of the all-affecting nature of a personal crisis.

It was in remembering back to recovery of consciousness in intensive care that I came to the notion of sentience. That moment also represented final return to consciousness from the cardiac arrest: realisation that I was, indeed, alive.

I had gone into theatre knowing that I might not come out alive. At the moment of reactivating consciousness, I simply remember a sensory experience of whiteness, whether from something external or from within, like a screen coming to life, making me wonder, ‘Where am I?’ Then I recalled being told, as I was being wheeled into theatre, that after the operation I would be taken to intensive care. So … that must be where I was. Which meant I must be alive. ‘That's nice’, I thought.

The trite words reflected a two-dimensional state of awareness: the cognitive intensity of the moment precluding reflective consciousness. Meanwhile, the felt question ‘Where am I?’ presupposed, at a purely sentient level, trust in the fact of my own existence.

Can there be any form of consciousness without that fundamental level of existential trust? How does simple animate matter relate to the phenomenon of consciousness? From a position of faith, philosophical discussions of the nature of mind and consciousness in relation to matter, in particular the brain, are reminiscent of pre-scientific attempts to locate the seat of the soul. Confidence in one's own existence seems to me now, at a distance from intensive care, the pre-condition of any capacity for ideation; a synergy of different aspects of being that, if it can be conceptualised at all, would require so to speak quantum rather than Newtonian understanding.

## The body and mystery

Jesus wept. This manifestation of divine vulnerability precedes the raising of Lazarus from the dead (*John 11.1–44*). In hospital, more than once in emotional shock I pulled the bedding over my head and cried. When the Muslim chaplain, whom I knew through interfaith work in Newham, visited, I cried ‒ and apologised. What is the power and shame of tears? This purely physical ‘welling up’, a universal human experience, brings what is highest and deepest within us unavoidably to consciousness.

For over a year after leaving hospital, several times a day, without warning, with no conscious emotional desire to weep, I experienced a kind of hyperventilation like a child sobbing. Initially these spasms felt consistent with an underlying emotional state; but as they continued even after my emotional condition had stabilised, it occurred to me my body held memories that consciousness had either successfully processed or, as a result of the anaesthetic, never directly experienced. My sentient being had suffered more trauma than my ‘self’.

Although I had no near-death experience during cardiac arrest, my ‘conversion’ over 30 years previous had been precipitated ‒ the details are beyond the scope of this article ‒ by a frightening out-of-body experience in which I saw my body as a *thing* separate from me. The thing was discarded and I felt (as I articulated it at the time) that I had ‘jumped into my own body’, a new body, the true me.

Sentience may affect us in ways we cannot be conscious of, rather as cancer or medication are already at work before symptoms manifest. It may at some point become empirically explorable. Yet, more significantly for whole-person understanding, we already know it as mystery. Mystery can become glib, just as certainty, incurious about its own nature, can foreclose on open-minded experience; but as the liminal zone of our being, it transcends apprehension, interfaces with the transcendent … guiding the anaesthetist's hunch, informing the chaplain's smile.

In hospital after my operation a young doctor appeared one day: ‘You've been given a second life’, he said, hurrying off. I suspect it was he who had resuscitated me. The distinction between a second life (of extended mortality) and new life (qualitatively changed spiritual life) in Christ became crucial for me. Lazarus was not raised to immortality, and my bodily survival was a medical success story. So what?

My first near-death event was, in terms of *life*, more significant than the second; yet the quasi-miracle of physical survival years later brought life alive to me again. A vehicle of agony and abuse, delight and wonder, even at its most basic level of sentience the body is to the eyes of faith a sign (theologically, a sacrament) of a quite other order of being: a new mortality that is eternal, in tune with angelic intelligence (the substantiality of inexpressible communications), and destined for glory (the holistic experiential, philosophical and ethical ground of dignity of every human life).

In physically ingesting the body of Christ in the bread of the Eucharist we participate in the Body that is the Church ‒ a communion of minds and affections, vision and hope, compassion and concelebration, transcending its own fatal debilities. TPN sustains my second life, but it is the *mystery* of the body, new life, that makes that life worth living.

## Concluding reflections on care

In risking crossing the clinical boundary to address me personally, the anaesthetist transformed the crisis for me. The prayer with Brother Julian meant that I went into a dangerous operation with a sense of complete preparedness. But had I not myself known about chaplaincy, this care would not have been offered.

As a patient I was also conscious of the emotions and energy levels of carers ‒ professionalism cannot eradicate human relationship. I was touched and intrigued by the young doctor's visit, and longed for a chance to thank him properly.

Patients are the *objects* of medical professionals’ care. We are in their power: most of the time I experienced this as beneficent objectivity. Equally, however, I felt both we and they were caught in an under-resourced system that claims too much, creating depersonalising reification. COVID-19-era precautions will presumably make it even harder to maintain the personal relational care I found, and still find, so vital to well-being.

## About the author

**James Ramsay** is a Priest with permission to officiate within the Diocese of Norwich, Church of England, UK.

